# Supplementary material for: Predicting stop codon reassignment improves functional annotation of bacteriophages
Source: bioRxiv. 2023 Dec 19:2023.12.19.572299. Preprint. [Version 1] doi: 10.1101/2023.12.19.572299 (PMC10769273; doi:10.1101/2023.12.19.572299)
Supplement: Supplement 2 [file NIHPP2023.12.19.572299v1-supplement-2.pdf]

bioRxiv preprint doi: https://doi.org/10.1101/2023.12.19.572299; this version posted December 19, 2023. The copyright holder for this preprint (which was not certified by peer review) is the author/funder, who has granted bioRxiv a license to display the preprint in perpetuity. It is made available under aCC-BY 4.0 International license.

Supplementary Methods

Datasets

A multifasta file of phage genomes was downloaded from INPHARED (https://github.com/RyanCook94/inphared; September 2023)[6]. Stop codon reassignment of INPHARED genomes was predicted using Prodigal-gv v2.11.0 (https://github.com/apcamargo/prodigal-gv), a fork of Prodigal written to improve viral gene calling[8]. Those predicted to use translation table 4 or 15 were retained for downstream analysis.

The Unified Human Gut Virome Catalog (UHGV) was filtered for high quality and complete vOTUs deemed to be a “high confidence” virus and predicted to use either translation table 4 or 15 (https://github.com/snayfach/UHGV). Stop codon reassignment had already been predicted for UHGV vOTUs using Prodigal-gv and is available in the UHGV metadata.

Prokka

A fork of Prokka v1.14.5[11] was written that incorporates an initial stage of ORF prediction using Prodigal-gv v2.11.0 (https://github.com/apcamargo/prodigal-gv)[8]. A first gene calling step is used to infer the genetic code most likely adopted by the genome, then the predicted genetic code is used to perform the translation FASTX::Seq, which we updated to accept code 15 (metacpan.org/pod/FASTX::Seq)[16]. The code for this is available at (github.com/telatin/metaprokka). We included publicly available HMMs of the PHROGs database in our Prokka-gv annotations (http://s3.climb.ac.uk/ADM_share/all_phrogs.hmm.gz)[17]. The fork is installable from Bioconda as ‘metaprokka’.

Pharokka

Pharokka v1.5.0[12] was updated to include support for pyrodigal-gv implementing pyrodigal-gv as a gene predictor. This is specified by using ‘-g prodigal-gv’ when running Pharokka. The updated code is available on GitHub (https://github.com/gbouras13/pharokka). Pharokka uses tRNAscan-SE for predicting tRNAs[14].

bioRxiv preprint doi: https://doi.org/10.1101/2023.12.19.572299; this version posted December 19, 2023. The copyright holder for this preprint (which was not certified by peer review) is the author/funder, who has granted bioRxiv a license to display the preprint in perpetuity. It is made available under aCC-BY 4.0 International license.

Statistical Analyses and Data Visualisation

To test for significance in differences of results, a simple paired T test was performed in R v4.2.2[18] and P-values were adjusted using the Benjamini-Hochberg procedure[19]. Figure 1 was produced using ggplot2 v3.4.2[20].

bioRxiv preprint doi: https://doi.org/10.1101/2023.12.19.572299; this version posted December 19, 2023. The copyright holder for this preprint (which was not certified by peer review) is the author/funder, who has granted bioRxiv a license to display the preprint in perpetuity. It is made available under aCC-BY 4.0 International license.

Supplementary Results

Prokka-gv Annotations

For Prokka-gv, the largest differences were observed for sequences predicted to use translation table 15, for which Prokka-gv increased the median gene length (median of per genome medians) from 276 to 396 bp for UHGV sequences (43.5% increase), and from 309 to 483 bp for INPHARED sequences (56.3% increase). This was also reflected in an increase of median coding capacity from 66.6% to 86.7% for UHGV, and from 69.2% to 87.3% for INPHARED. As it is commonly used as a phylogenetic marker for bacteriophages, we investigated how commonly the major capsid protein (MCP) could be identified with and without predicted stop codon reassignment[15]. For sequences predicted to use translation table 15, the MCP could be identified on 382/715 (53.4%) sequences with Prokka and this was marginally increased to 386/715 (53.9%) with Prokka-gv.

When investigating the sequences for which translation table 4 was predicted, a substantial increase was also observed for UHGV sequences, with Prokka-gv increasing median median gene length from 319 to 460 bp (44.2%), resulting in an increase of coding capacity from 78.4% to 91.4%. However, the same was not observed for INPHARED sequences predicted to use translation table 4. These sequences observed a modest increase in median median gene length from 573 to 584 bp (1.8%) for Prokka-gv. Median coding capacity was not increased with Prokka and Prokka-gv both obtaining 86.2%.
